# Mannose-modified erythrocyte membrane-encapsulated chitovanic nanoparticles as a DNA vaccine carrier against reticuloendothelial tissue hyperplasia virus

**DOI:** 10.3389/fimmu.2022.1066268

**Published:** 2023-01-04

**Authors:** Yangyang Feng, Feng Tang, Sheng Li, Daiyan Wu, Qianqian Liu, Hangyu Li, Xinnan Zhang, Ziwei Liu, Linzi Zhang, Haibo Feng

**Affiliations:** ^1^ College of Animal Husbandry and Veterinary Medicine, Southwest Minzu University, Chengdu, China; ^2^ Key Laboratory of Ministry of Education and Sichuan Province for Qinghai-Tibetan Plateau Animal Genetic Resource Reservation and Utilization, Southwest-Minzu University, Chengdu, China

**Keywords:** reticuloendothelial virus bionic, chitosan, erythrocyte membrane, mannose modification, delivery system

## Abstract

**Introduction:**

The erythrocyte membranes used in nanovaccines include high membrane stability, long circulation life, adaptability and extremely good bio compatibility. Nanoparticles encapsulated by erythrocyte membranes are widely used as ideal drug delivery vehicles because of their high drug loading, long circulation time, and excellent biocompatibility. The mannose modification of delivery materials can help target mannose receptors (MRs) to deliver antigens to antigen-presenting cells (APCs).

**Methods:**

In this study, the antigen gene gp90 of avian reticuloendotheliosis virus (REV) was encapsulated with carboxymethyl chitosan (CS) to obtain CSgp90 nanoparticles, which were coated with mannose-modied fowl erythrocyte membranes to yield CS-gp90@M-M nanoparticles. The physicochemical characterization and immune response of the CS-gp90@M-M nanoparticles were investigated *in vitro* and *in vivo*.

**Results:**

CS-gp90@M-M nanoparticles were rapidly phagocytized in vitro by macrophages to induce the production of cytokines and nitric oxide. In vivo, CS-gp90@M-M nanoparticles increased cytokine levels, the CD4+/8+ ratio, REV-specific antibodies in the peripheral blood of chicks, and the mRNA levels of immune-related genes in the spleen and bursa of immunized chicks. CS-gp90@M-M nanoparticles could be targeted to lymphoid organs to prolong the retention time of the nanoparticles at the injection site and lymphatic organs, leading to a strong, sustained immune response. Moreover, the CS-gp90@M-M nano-vaccine showed a lasting immunoprotective effect and improved the body weight of chicks after the challenge.

**Conclusion:**

Overall, CS-gp90@M-M nanoparticles can be used in vaccine designs as an effective delivery carrier with immune response-enhancing effects.

## 1 Highlights

1. The mannose receptor targeted Biomimetic REV nucleic acid vaccine (CS-gp90@M-M) were constructed for the first time.
2. CS-gp90@M-M significantly promoted the immune activity of macrophage in vitro.
3. CS-gp90@M-M could effectively activate the immune system and offer immune protection against REV challenge in chicks.
4. CS-gp90@M-M can slow release in injection site and stay longer in the immune organs in chicks.
5. CS-gp90@M-M could effectively promote humoral and cellular immune responses.

## 2 Introduction

Avian reticuloendothelial hyperplasia virus (REV) has a worldwide prevalence and chicks, ducks, turkeys, and other poultry are sensitive to this virus (1). REV can cause acute reticular cell tumor formation dwarfism syndrome, and chronic tumors of lymphoid and other tissues. REV infection also causes immunosuppression and decreases the immune efficacy of other vaccines, leading to infected chickens that are prone to secondary bacterial and other viral infections, with significant economic losses to the poultry industry. Currently, there is no effective commercial vaccine available to prevent this disease (2, 3).

Nanoparticle carriers show immense potential in vaccine development (4, 5). Bionic nanoparticles wrapped around cell membranes are a drug delivery vehicle with the combined advantages of synthetic nanoparticles and natural cell membranes. As highly abundant circulating cells in vertebrates, erythrocytes perform the special function of capturing certain pathogens in the blood and presenting them to the immune cells in secondary immune organs. Thus, erythrocytes are a kind of natural “innate carrier” with many unique advantages in drug delivery (6). Erythrocyte membranes can be used as an encapsulating material to decorate nanoparticles as delivery vehicle systems with the advantages of long circulation, high membrane flexibility and stability (7), good biocompatibility (8), the retention of basic cellular functions, and functional groups that can be modified on the membrane surface (9). Owing to these special advantages, the use of erythrocytes as biomimetic drug nanomaterials to treat diseases and modulate immune function shows great potential.

In gene therapy, the structural characteristics of the target gene and the physiological barrier function of the body make it difficult to effectively target specific cells and organs This process can be completed with the help of vectors (10). Chitosan is a nontoxic and harmless nano-material prepared from the deacetylation of natural polysaccharides. Positively charged chitosan can combine with negatively charged drugs to increase drug stability (11, 12). Currently, the main drawback of chitosan as a gene delivery system is its limited solubility under physiological conditions and low effective transfection efficiency. Thus, methylated carboxymethyl chitosan (C_20_H_37_N_3_O_14_) is used as a cell membrane core to wrap nanoparticles. Carboxymethyl chitosan can be obtained under alkaline conditions. The substitution of carboxymethyl will occur on both -OH and -NH to form O-carboxymethyl and N-carboxymethyl chitosan. Carboxymethyl chitosan is not only soluble in water because it is a sodium carboxylate salt, but also because the introduction of carboxymethyl destroys the secondary structure of the chitosan molecules, so their crystallinity is greatly reduced and the material almost becomes amorphous (13). CS has higher water solubility at physiological pH, facilitating nanoparticle synthesis and rendering it more suitable for drug delivery and biological imaging (14).

Mannose receptors (MRs) belong to the C-type lectin receptor superfamily and are widely distributed on the membranes of dendritic cells (DCs) and macrophages. MRs play a crucial role in pathogen recognition and homeostasis. MRs can also recognize mannose residue–conjugated adjuvants and drug-delivery materials. Many studies have been designed to improve the immune response of cell membrane–encapsulated nanoparticles, where mannose was commonly used as the targeted ligand for specific targeting to antigen-presenting cells (APCs) (15).

We hypothesized that erythrocyte membrane encapsulation and mannose-targeted modification could enhance the immunogenicity of nanoparticles, while chitosan could enhance the gene-carrying capacity of nanodrug delivery systems. Therefore, the current study aimed to investigate the immune response of mannose-modified erythrocyte membrane-coated chitosan bionic nanoparticles and evaluate their potential application in the development of poultry vaccines.

## 3 Materials and methods

### 3.1 Materials

Phosphatidyl ethanolamine-polyethylene glycol-mannose (DSPE-PEG-Mannose) was obtained from Shanghai Yanyi Biology Co., Ltd. (Shanghai, China). Carboxymethyl chitosan (deacetylation degree: 85%; MW:600 kDa) was purchased from Xi’an Baichuan Biotechnology Co., Ltd. (Xi’an, Shanxi, China). The nitric oxide (NO) test kit, lipophilic fluorescent DiD dye, 4’,6-diamidino-2-phenylindole (DAPI) DNA fluorescent dye, the chicken lymphocyte separation kit, and Methylthiazolyldiphenyl-tetrazolium bromide (MTT) were procured from Beijing Solarbio Technology Co., Ltd. (Beijing, China). The REV antibody kit, interferon (IFN)-γ, and interleukin (IL)-4 enzyme-linked immunosorbent assay (ELISA) kits were obtained from Jiangsu Meimian Industry Co., Ltd. (Nanjing, Jiangsu, China). Mouse anti-chicken CD4^+^-FITC and CD8^+^-PE antibodies were procured from eBioscience (San Diego, CA, USA). Avian reticuloendotheliosis virus (CVCC: CVCCAV107) was purchased from the Chinese Veterinary Microbial Strain Preservation and Management Center.

### 3.2 Preparation of CS-gp90@M-M nanoparticles

The pTT5-gp90 recombinant plasmid (Nanjing, Jiangsu, China) was prepared according to the plasmid extraction kit instructions and purified using PEG8000 following a previously published study (16). Briefly, 250 μg/mL of carboxymethyl chitosan and 100 μg/mL of pcDNA-gp90 plasmid solution were mixed, stirred for 30 s, and stored at 25 °C for 1 h (17). The collected blood was centrifuged at 1500 ×*g* for 3 min at 4°C, and the serum was removeThe erythrocytes were soaked for 20 min in an osmotic phosphate-buffered saline (PBS) ice bath and collected by centrifugation. After 3 washes, the erythrocyte membranes were resuspended in PBS and mixed with CS-gp90 for 18 h at 4°C (18). The solutions were combined and passed 20 times through a liposome extruder with a 200-nm pore diameter filter membrane, and then centrifuged to remove excess erythrocyte membranes. After stirring for 1 h, 0.1 mg/mL of DSPE-PEG-MAN solution was added to insert mannose into the erythrocyte membranes to yield mannose-embedded chicken erythrocyte membranes.

### 3.3 Physicochemical properties of CS-gp90@M-M nanoparticles

Agarose gel electrophoresis was used to detect whether the *gp90* gene was correctly inserted into the plasmid. The ultra-microstructure of the erythrocyte membrane–encapsulated nanoparticles (CS-gp90@M-M) was observed using transmission electron microscopy (TEM) (FEI, Tecnai G2 F20 S-TWIN, CA, USA) and the images were photographed. DSPE-PEG-Man, CS-gp90@M, and CS-gp90@M-M spectra were obtained using Fourier-transform infrared (FT-IR) spectroscopy (8400S, Shimadzu Co., Nakagyo-ku, Kyoto, Japan). IR spectra were recorded and analyzed in the range of 400 to 4000 cm^−1^. CS-gp90@M and CS-gp90@M-M samples were mounted on glass slides and analyzed using Raman spectroscopy (Horiba, LabRAM, France) at 532 nm in the range of 1000 – 2000 cm^−1^. The particle size and polymer dispersity index (PDI) of the nanoparticles were determined using a laser particle-size analyzer. Various nanoparticles were simultaneously placed in a potential cup to measure their zeta potential. A standard curve of a CS-gp90 solution was constructed, and the absorbance of the samples was measured at 470 nm. The CS-gp90 concentration in the supernatant was determined using the standard curve. The adsorption rate and drug-loading rate were calculated using formulas (1) and (2) (19).


(1) $$\begin{array}{c} Drug-loading\;rate\;(\%)\\ \;=\;(CS-gp90\;mass–CS\\ \;-gp90\;mass\;in\;the\;supernatant)\;/\;carrier\;total\;mass\\ \;\times \;100\% \end{array}$$

(2) $$\begin{array}{c} Entrapment\;efficiency\;(\%)\\ \;=\;(CS-gp90\;mass–CS\\ \;-gp90\;mass\;in\;the\;supernatant)\;/\;drug\;dosage\\ \;\times \;100\% \end{array}$$

### 3.4 *In vitro* experiments

#### 3.4.1 Cytotoxicity of CS-gp90@M-M nanoparticles

*In vitro* cytotoxicity tests were performed using the MTT method (20). Briefly, 2 × 10^4^/mL HD11 cells were added to 96-well cell culture plates and treated with various concentrations of CS-gp90@M and CS-gp90@M-M nanoparticles at 40 °C. After 24 h, 100 μL of medium and 10 μL of MTT (5 mg/mL) were added for 4 h and the supernatant was aspirated after a purple precipitate was formed. Next, 150 μL of dimethyl sulfoxide was added to each well. The absorbance of each well was determined using a microplate reader (Bio-Rad, iMark, Hercules, CA, USA) at 570 nm.

#### 3.4.2 Uptake of CS-gp90@M-M nanoparticles by HD11 cells

Cell uptake assay. HD11 cell solution (1 mL) at a concentration of 8 × 10^4^ cells/mL was added to a 24-well plate, and 50 μL of DiD-stained CS-gp90@M and CS-gp90@M-M (200 μg/mL) nanoparticles were filtered. After incubation for 8 h in the dark, the culture medium was discarded, and the cells were washed twice with PBS, stained with DAPI dye, and washed twice for 20 min. A drop of anti-fluorescence quenching agent was added to the cells on a clean slide. The slide was analyzed using confocal laser scanning microscopy (CLSM) (ZEISS, Oberkochen, Germany).

Quantitative test to determine cell uptake activity. DiD-stained CS-gp90@M (50 μL) and CS-gp90@M-M (200 μg/mL) nanoparticles were incubated with 5 × 10^5^ HD11 cells in a Petri dish for 8 h. The intracellular fluorescence intensity of DiD was measured using a small animal *in vivo* imager (Perkin Elmer IVIS LuminaIII, Waltham, MA, USA).

#### 3.4.3 NO secretion and iNOS mRNA expression

Cells in the logarithmic growth phase were seeded in a 24-well cell culture plate at a density of 1×10^6^ cells/mL. Based on the concentration of the plasmid, 100 μg/mL of the nanoparticle solutions prepared using 1 mL of DMEM basic culture medium (PBS group, gp90 plasmid group, CS-gp90 nanoparticle group, CS-gp90@M nanoparticle group, CS-gp90@M-M nanoparticle group) and the positive lipopolysaccharide (LPS) control (1 μg/mL) were cultured for 24 h. The nitric oxide (NO) content was determined using an NO detection kit (Nanjing, China). The absorbance was determined using a microplate reader at 540 nm, and the NO concentration was calculated using the standard gradient curve. Total RNA was extracted from each group of cells using an RNA extraction kit for cDNA synthesis. The level of inducible nitric oxide synthase (iNOS) mRNA was analyzed using a real-time polymerase chain reaction detection system (CFX96, Bio-Rad).

### 3.5 *In vivo* experiments

#### 3.5.1 Animal immunization

Sixty 1-day-old Dehua black chicks without REV were purchased from the Chengdu animal market in Sichuan Province and fed adaptively for 3 days. The study was conducted according to the guidelines of the Declaration of Helsinki and approved by the Institutional Ethics Committee of Southwest Minzu University and the Institutional Animal Care and Use Committee of Southwest Minzu University (IACUC approval No.: IACUC-20211012-21). The chicks were randomly divided into 5 groups (PBS group, gp90 plasmid group, CS-gp90 nanoparticle group, CS-gp90@M nanoparticle group, and CS-gp90@M-M nanoparticle group). Based on entrapment efficiency and drug loading, the drug was injected into the leg muscle at a dose of 1 μg/g body weight. Chicks in the PBS group were administered an equal volume of sterilized PBS in the same manner, and booster immunization was performed on day 14 after the first immunization. Chicks from the different groups were raised in separate cages. Blood from the subtalar vein was collected every week and used to determine various indices after anticoagulation.

#### 3.5.2 Antibody ELISA

Serum was obtained from the peripheral blood of immunized chicks collected at 0 – 5 weeks, and serum antibody titers were determined using an enzyme-linked immunosorbent assay (ELISA) antibody detection kit for REV. The optical density (OD) of the samples was determined at 450 nm. Antibody titers were calculated based on the statistical analysis of the data (S/P value, S represents sample pore OD value; P represents positive hole OD value).

#### 3.5.3 Cytokine assays

After 21 days following the first immunization, 3 chicks in each group were randomly selected and blood was collected from the inferior wing vein. The serum was separated and stored at -20 °C until further use. Serum cytokine levels of IFN-γ and IL-4 were determined using the corresponding ELISA kits following the manufacturer’s instructions.

#### 3.5.4 T lymphocyte subsets

On the 21st day after primary immunization, 3 chicks per group were randomly selected and 1 mL of blood was collected. Isolated lymphocytes (2×10^6^ cells/mL; 50 μL) were stained using mouse anti-chicken CD3^+^-allophycocyanin (APC), CD4^+^-fluorescein isothiocyanate (FITC), and CD8^+^-phycoerythrin (PE) fluorescent antibodies. The percentage of CD4^+^ and CD8^+^ T lymphocytes in T cells was measured by florescence-activated cell sorting (FACS; BD FACSVerseTM, CA, USA), and the ratio of CD4^+^/CD8^+^T cells was calculated (21).

#### 3.5.5 Detection of immune-related molecules by qRT-PCR

The mRNA level of Interferon induced with helicase C domain 1 Protein (IFIH1), interferon regulatory factor 7 (IRF7), toll-like receptor (TLR)3, signal transducer and activator of transcription 1 (STAT1), viperin, IFN-γ, and IL-4 were determined by qRT-PCR. An RNA extraction kit was used to extract RNA from the spleen and bursa tissues of chicks on the 21st day of the experiment. Spare tissues (30 mg) from each group were pulverized in a mortar and quickly ground with liquid nitrogen. The remaining steps were the same as those listed in Section 2.4.3.

#### 3.5.6 Delivery kinetics of the constructed nano-vaccines

After adaptive feeding, chicks were randomly selected and intramuscularly injected with one of the DiD-stained nano-vaccines (CS-gp90@M and CS-gp90@M-M nanoparticles) at a dose of 10 μg/g body weight. Biomimetic nanoparticles labeled with DiD at different time points (0, 6, 12, 24, 48, and 72 h) after immunization were detected using a PerkinElmer IVIS Lumina III *in vivo* imaging system. To quantify the biomimetic nanoparticles in different tissues and organs, we used a small animal *in vivo* imaging system to determine the fluorescence intensity of chicken organs in an *ex-vivo study*.

#### 3.5.7 Protective effects of CS-gp90@M-M nanoparticles against REV infection

One hundred and eighty chicks were randomly divided into 5 groups, and 36 chicks in each group were randomly divided into 3 batches, with 12 chicks per batch. A challenge test was performed on the 3rd, 15th, and 30th days after vaccination. Chicks were intraperitoneally infected with a 100-fold dilution of the 50% tissue culture infectious dose (TCID50 REV (TCID50 = 10-4.62/0.1 mL) and then reared in isolation. Cumulative mortality was calculated 21 days after the challenge, and the changes in the body weight of the chicks in each group were determined at the same time.

#### 3.5.8 Observation of the toxicity of CS-gp90@M-M nanoparticles

On the 28th day of the experiment, 3 chicks from each group were randomly selected, and their kidneys, spleens, lungs, livers, and hearts were collected. Paraformaldehyde solution (4%) was used to fix the collected samples. Tissues were embedded in paraffin, sectioned, stained with hematoxylin and eosin (H&E), sealed, and observed using microscopy.

### 3.6 Statistical analysis

The results are presented as the mean ± standard deviation (n=3). The data were analyzed using SPSS 22 (SPSS, Chicago, IL, USA). Differences between the groups were analyzed using the Duncan test and (Least-Significant Difference) LSD multiple comparisons tests. A *P*-value of*<* 0.05 was considered significantly significant.

## 4 Results

### 4.1 Characterization

#### 4.1.1 The construction of CS-gp90

**Figure 1:**
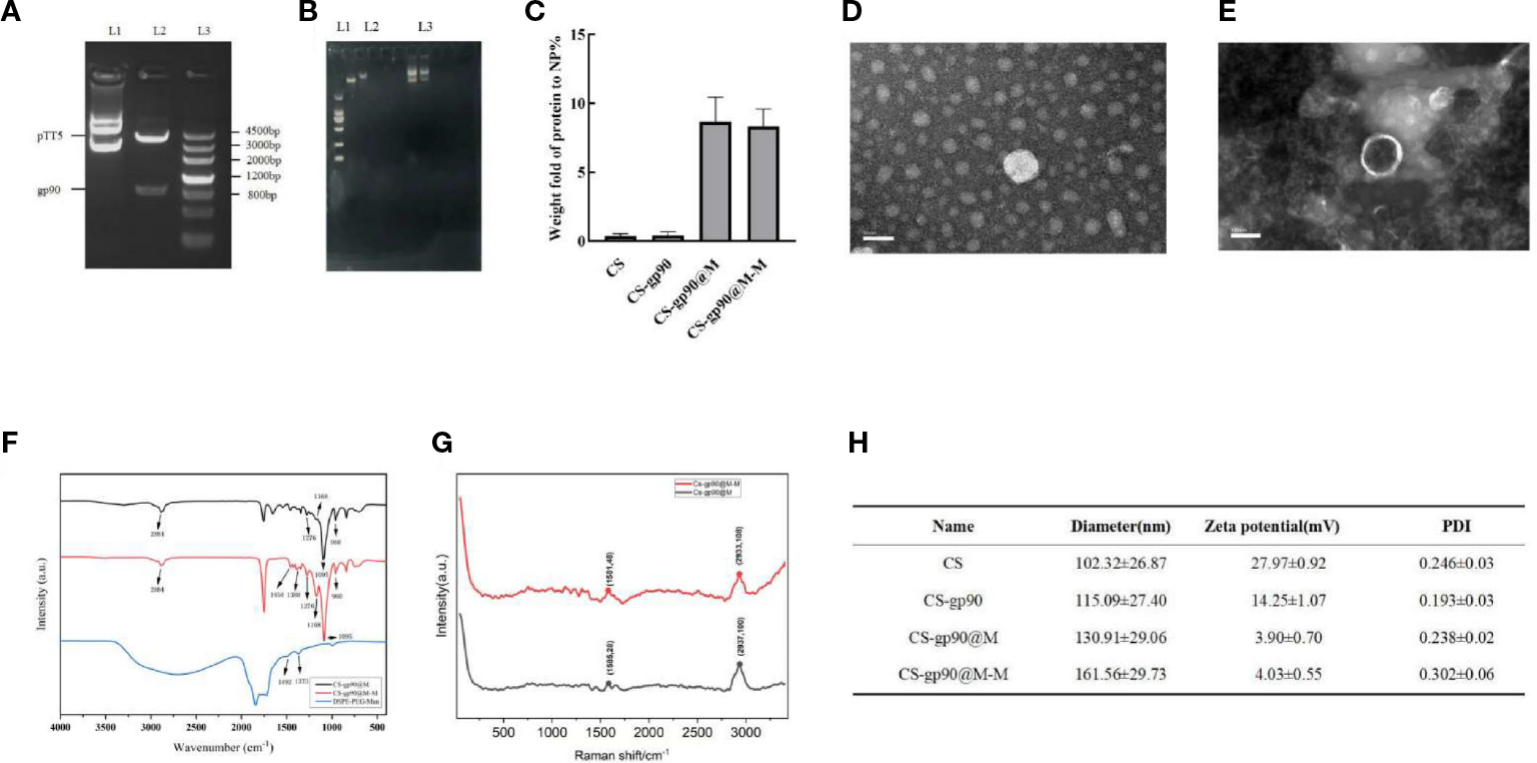
Physicochemical properties of the constructed nanoparticles. **(A)** *gp90* gene identification L1: pTT5 plasmid DNA; L2: Digested with HindIII/SmaI. L3: DNA marker. **(B)** The complexation and condensation of CS-gp90. **(C)** BCA assay to determine the protein content of the CS, CS-gp90, CS-gp90@M, and CS-gp90@M-M samples. **(D, E)** TEM image of CS-gp90 and CS-gp90@M-M nanoparticles. **(F)** Infrared spectra of bionic nanoparticles. **(G)** Raman spectra of bionic nanoparticles; **(H)** Hydrodynamic sizes, zeta potentials and PDI of the nanoparticles.

In this study, the products of digested plasmid DNA and HindIII/SmaI analyzed using 1% agarose gel electrophoresis showed obvious bands around 950 bp, consistent with the expected results, indicating that the *gp90* gene was correctly inserted into the pTT5 plasmid (**Figure 1A**). The complexation and condensation of DNA in polyplexes with chitosan was studied by using agarose gel electrophoresis. The results demonstrated that the migration speed of CS-gp90 in electrophoresis was significantly slower than that of gp90, indicated that the complexation and condensation of DNA and chitosan in the polymer increase the molecular weight of gp90 (**Figure 1B**).

#### 4.1.2 Membrane proteins analysis

Membrane proteins are the main component of erythrocyte membranes. Erythrocyte membrane-coated nanoparticles (CS-gp90@M and CS-gp90@M-M) showed a significant increase in protein content compared to exposed nanoparticles. resulting in and the protein content of both types of nanoparticles was similar (**Figure 1C).**

#### 4.1.3 TEM morphological characteristics

The micro morphology of CS-gp90 NPs was observed as round or oval solid particles with uniform distribution and no aggregation (**Figure 1D**). The CS-gp90@M-M nanoparticles were round and spherical. A layer of red cell membranes was wrapped around the nanoparticles, and their surface was shell-like erythrocyte membranes with a typical shell-core structure (**Figure 1E**).

#### 4.1.4 FT-IR spectroscopy and raman spectrum analysis

The FT-IR infrared spectroscopy results are shown in **Figure 1F**. The FT-IR spectra of the mannose, CS-gp90@M, and CS-gp90@M-M were determined during the 4000 to 400 cm^−1^ range, and the results are shown in **Figure 1E**. The FT-IR spectrum of CS-gp90@M-M exhibits all the property peaks of CS-gp90@M, including a broadly intense stretched peak in the 3200 to 3600 cm^−1^ regions representing to hydroxyl and amino absorption peak stretching vibrations, and the specific absorption peak of methyl and methylene stretching vibrations was detected in the 2884 cm^−1^ region. A peak between 1500 and 1600 cm^−1^ indicated C=O asymmetric stretching vibrations. The absorption peaks around the 900 to 1200 cm^−1^ region is the C–O–C stretching vibration absorption corresponding glucose characteristics. Furthermore, the asymmetric stretching peaks in the 1276, 1168, 1095, and 960 cm^−1^ regions as the same absorption peaks as observed in CS-gp90@M. The other absorption peaks, in the 1454 and 1388 cm^−1^ regions, exhibited two absorption peaks of mannose. The results show that the CS-gp90@M-M obtained some characteristics of mannose, revealing that the mannose modification was successful (22). As there are no functional groups of mannose in simple erythrocyte membranes, it could be concluded that mannose-targeted erythrocyte membranes were successfully synthesized. This finding was also confirmed based on the characteristic peak shift in the Raman spectrum. The erythrocyte membrane nanoparticles were characterized using transmission electron microscopy (**Figure 1G**).

#### 4.1.5 Particle size, zeta potential, and PDI analysis

However, compared to the expected particle size, the measured size was relatively small, likely due to drying and dehydration during TEM. Combined with the findings from transmission electron microscopy, the successful encapsulation of the nanoparticles in erythrocyte membranes was confirmed. After coating with the cell membranes, the diameter of the CS-gp90@M nanoparticles increased slightly. Moreover, the particle size of CS-gp90@M nanoparticles increased after DSPE-PEG-Man modification, likely due to the polyethylene glycol chain anchored on the nanoparticle surface. The zeta potential of the nanoparticles decreased after they were coated with erythrocyte membranes (**Figure 1H**).

### 4.2 Cell viability

**Figure 2:**
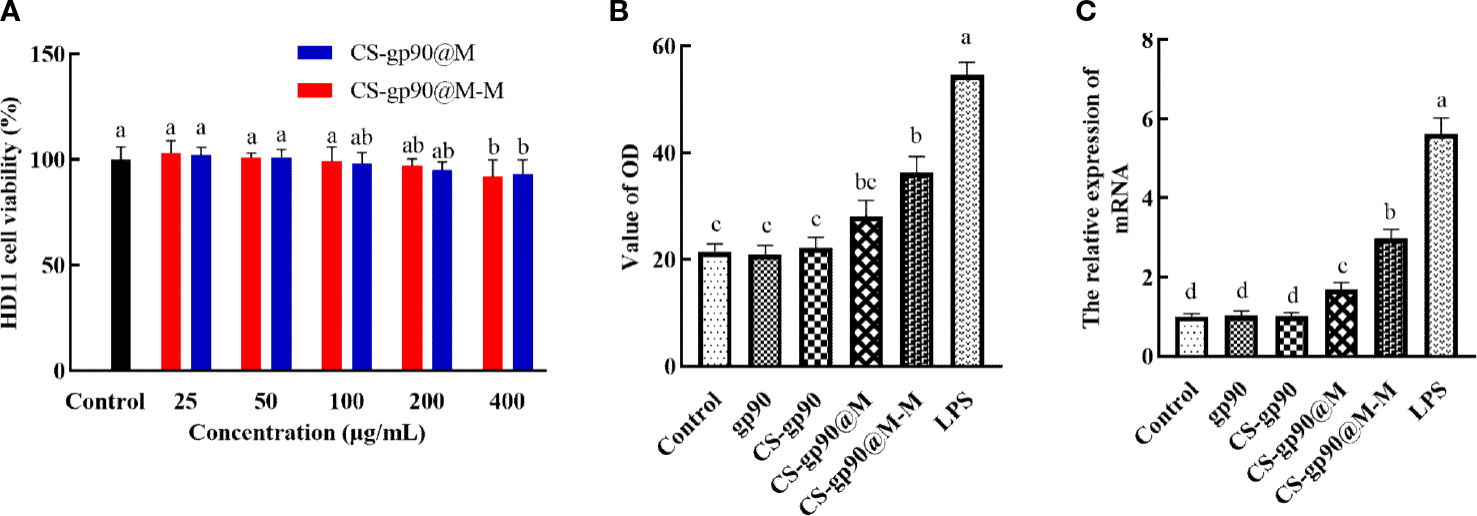
Toxicity and the immune-activating effect of CS-gp90@M-M nanoparticles on HD11 macrophages. **(A)** Effect of CS-gp90@M-M nanoparticles on macrophage viability, **(B)** NO release, and **(C)** iNOS mRNA levels. The different letters (a–d) designated as statistically significant differences (*P<0.05*).

As shown in **Figure 2A**, after incubation with different concentrations of CS-gp90@M and CS-gp90@M-M nanoparticles for 24 h, the cell survival rate was more than 90%. CS-gp90@M and CS-gp90@M-M nanoparticles were insignificantly toxic to HD11 macrophages, indicating the high biocompatibility of the biomimetic nano-vaccine and its potential for use in clinical settings.

### 4.3 NO production and iNOS mRNA expression

Activated macrophages inhibit the invasion of pathogens by releasing several important effector molecules including NO, which can regulate the functional activity of macrophages and mediate immune and inflammatory responses (23). iNOS is an enzyme that uses the oxidative stress (free radical) of NO to assist macrophages against pathogens in the immune system (24). iNOS generates peroxynitrite to produce NO through interactions with superoxide free radicals, which is considered to be the first immune defense response of the host against invading pathogens, especially intracellular pathogens. This reaction occurs only after macrophages are stimulated and activated, thus producing a large amount of NO (25). In the present study, a significant increase in NO release from HD11 macrophages was seen when CS-gp90@M-M nanoparticles were used (**Figure 2B**). The effect of CS-gp90@M-M nanoparticles on iNOS mRNA levels was determined by qRT-PCR, as seen in **Figure 2C**. CS-gp90@M-M nanoparticles significantly increased the mRNA level of iNOS in HD11 cells compared to the control, gp90, CS-gp90, and CS-gp90@M groups.

### 4.4 APC-targeting ability of the biomimetic nano-vaccine

**Figure 3:**
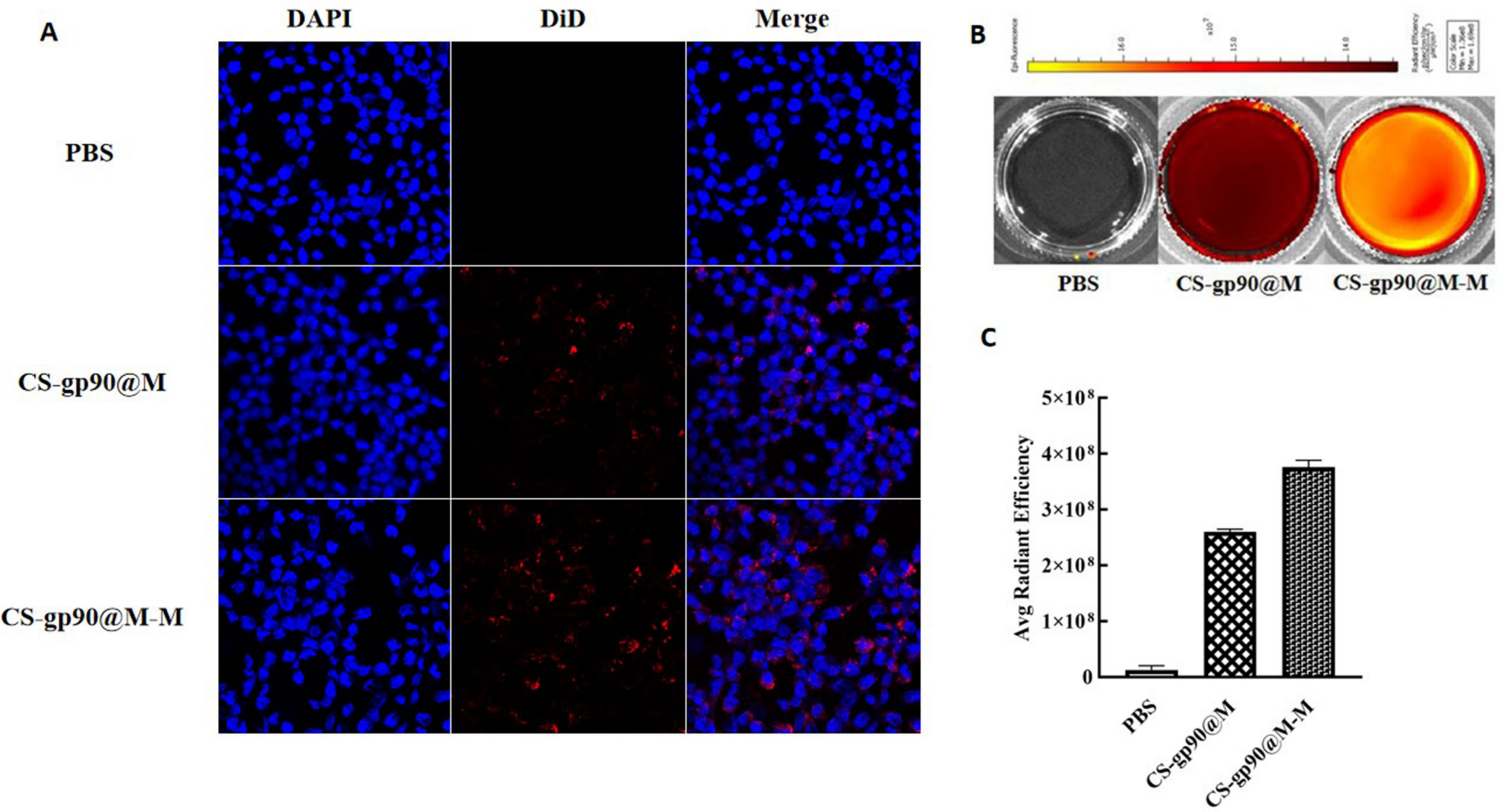
CS-gp90@M-M uptake by macrophages. **(A)** CLSM image of DiD-stained CS-gp90@M and CS-gp90@M-M nanoparticles up take by macrophages *in vitro*. Macrophage solution (8 × 10^4^ cells/mL) was added to a 24-well plate, and 50 μL of DiD-stained CS-gp90@M and CS-gp90@M-M (200 μg/mL) nanoparticles were added and incubated for 8 h. Then, the cells were stained with DAPI dye and washed twice for 20 min. The cells were mounted using glycerol (90%) and observed by CLSM. Blue fluorescence represents DAPI-stained nuclei and red fluorescence represents DID-stained CS-gp90@M and CS-gp90@M-M nanoparticles. **(B)** The uptake of CS-gp90@M-M nanoparticles by macrophages using an IVIS instrument. Macrophages were added to a 6-well plate and cultured for 24 h. DiD-stained CS-gp90@M (50 μL) or CS-gp90@M-M (200 μg/mL) nanoparticles were added to macrophages (5 × 10^5^) in a Petri dish for 8 h. The cells were collected and the intracellular fluorescence intensity of DID was determined using the IVIS instrument. The image shows the fluorescence intensity of the cellular uptake of CS-gp90@M and CS-gp90@M-M nanoparticles by macrophages. **(C)** The average radiant efficiency of the macrophage uptake of nanoparticles.

In both cellular immunity and humoral immunity, the treatment and expression of pathogenicity-related antigens through APCs is an important process (26). Therefore, the phagocytosis of the biomimetic nano-vaccine by APCs is important for immune responses and immune protection (27, 28). In the present study, CS-gp90@M-M and CS-gp90@M nanoparticles were stained with DiD and phagocytosis of the mannose receptor-targeting biomimetic nanoparticles by APCs was evaluated to investigate the targeting ability of the mannose receptor biomimetic nanoparticles. HD11 macrophages were incubated with stained CS-gp90@M or CS-gp90@M-M nanoparticles and analyzed by laser confocal microscopy. As shown in **Figure 3A**, compared to the CS-gp90@M group, the CS-gp90@M-M group demonstrated more red fluorescence–stained nanoparticles near the blue nucleus, suggesting that more CS-gp90@M-M nanoparticles were engulfed by the macrophages. Grafted mannose significantly improved the macrophage-targeting ability of the nanoparticles.

To quantitatively analyze the uptake of CS-gp90@M and CS-gp90@M-M nanoparticles by HD11 macrophages, cells and nanoparticles co-incubated in Petri dishes were analyzed using an animal *in vivo* imaging system (29). As shown in **Figures 3B, C**, the average fluorescence intensity of HD11 cells incubated with CS-gp90@M-M nanoparticles was higher than that of CS-gp90@M nanoparticles without mannose modification, suggesting that mannose modification promoted the phagocytosis of nanoparticles by macrophages.

### 4.5 Serum REV-specific antibody titers in chicks immunized with CS-gp90@M-M nanoparticles

**Figure 4:**
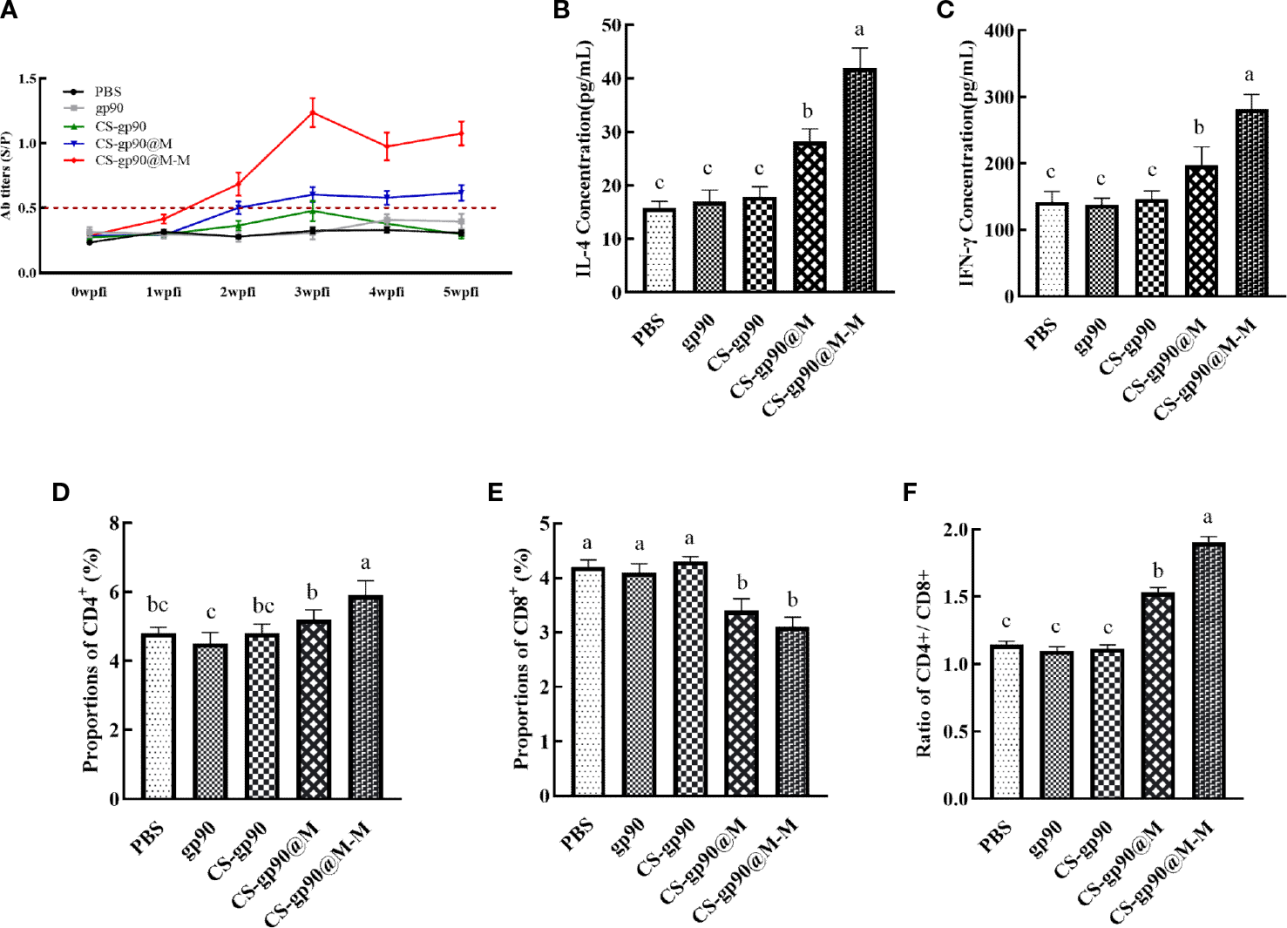
Effect of CS-gp90@M-M NPs on the antibody response, T lymphocyte phenotype, and cytokine production in chicks. Serum samples from chicks were collected on days 7, 14, 21, 28, and 35 for ELISA analysis after the first immunization. REV-specific IgG levels **(A)** were determined using ELISA. IL-4 **(B)** and IFN-γ **(C)** levels in the serum of chicks were determined using ELISA. CD4+ **(D)**, CD8+ **(E)**, and the CD4+/CD8+ T cell ratio **(F)** in peripheral blood were analyzed using FACS. The different letters (a–c) designated as statistically significant differences (*P<0.05*).

*In vivo* antibody titers are an important indicator of drug immunogenicity (30). As illustrated in **Figure 4**, the REV serum antibodies in the CS-gp90@M-M group increased rapidly, especially after the second week following booster immunizations. The antibody titer increased significantly, whereas the REV serum antibody titer of the other groups was relatively low and increased slowly. The antibody titer in the CS-gp90@M group was higher than that in the other 3 groups and the positive control. These results showed that mannose modification and encapsulation by erythrocyte membranes could dramatically increase REV-specific antibody titers.

### 4.6 Serum levels of cytokines in chicks immunized with the bionic nano-vaccine

Cytokines are a kind of protein or small molecule polypeptide that can transfer information between cells and has immune-regulating and other effects. Serum cytokine levels can reflect the immune response status in the body. As illustrated in **Figures 4B, C**, the serum levels of cytokines (IL-4 and IFN-γ) in the mice in the CS-gp90@M group were significantly greater than those in the mice in the CS-gp90, gp90, and PBS groups on day 21, indicating that erythrocyte membranes encapsulation could increase the activity of CS-gp90 NPs in inducing cytokine secretion in chicks and induce both Th1 and Th2-type immune responses. Serum IL-4 and IFN-γ levels in the CS-gp90@M-M group were remarkably higher than those in the CS-gp90@M, CS-gp90, gp90, and PBS groups on day 21, suggesting that CS-gp90@M-M NPs could increase both Th1 and Th2-type cytokine production.

### 4.7 Proportion of CD4^+^ and CD8^+^T lymphocytes, and the CD4^+^/CD8^+^ ratio

The proportion of CD4+ and CD8+ T lymphocytes and the CD4+/CD8+ ratio are key biomarkers that can reflect the function of T lymphocytes. As seen in **Figure 4**, compared to other groups, the proportion of CD8^+^ T lymphocytes in the CS-gp90@M-M group was notably decreased (*P<* 0.05), whereas the percentage of CD4^+^T cells and the CD4^+^/CD8^+^ ratio increased significantly (*P<* 0.05). The percentage of CD8^+^T lymphocytes in the CS-gp90@M group was significantly decreased (*P<* 0.05), whereas the percentage of CD4^+^ T cells and the CD4^+^/CD8^+^ ratio was significantly higher than those in the PBS, gp90, and CS-gp90 groups (*P<* 0.05). Treatment with both CS-gp90@M-M and CS-gp90@M NPs could partially improve the immune response by enhancing T lymphocyte subsets in the immunized chicks.

### 4.8 Expression of immune-regulating genes in chicks immunized with CS-gp90@M-M NPs

**Figure 5:**
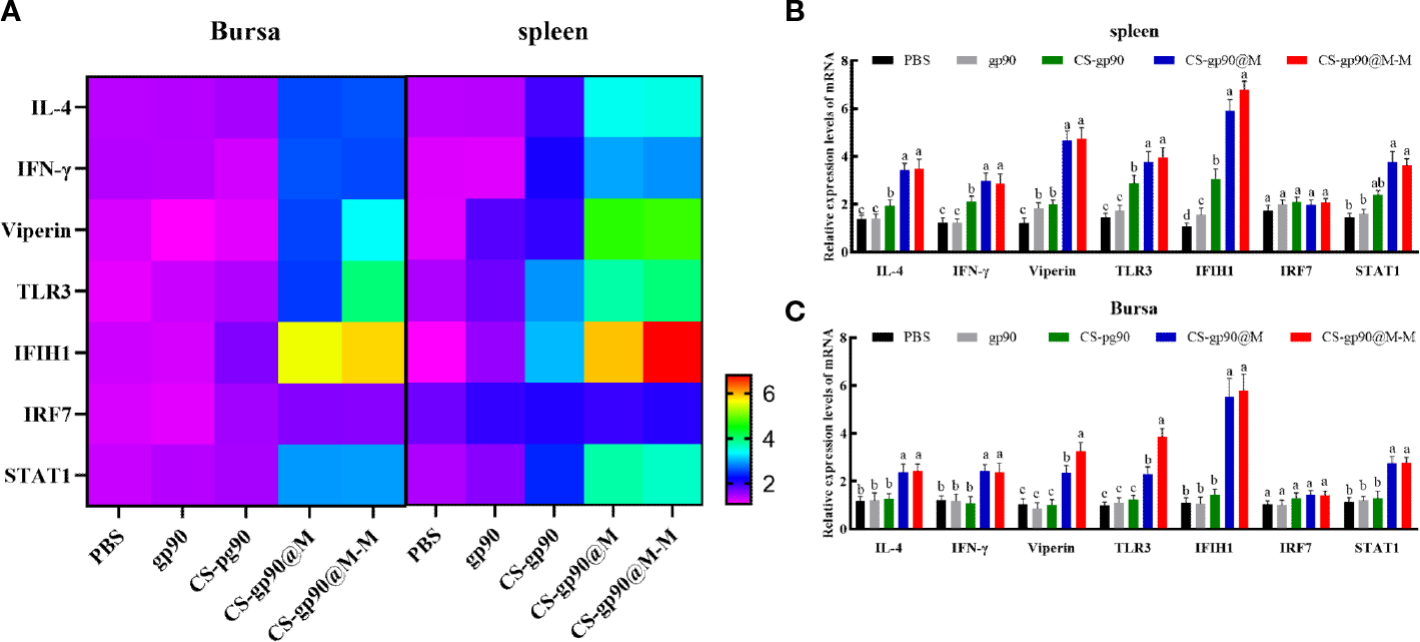
Effect of CS-gp90@M-M NPs on the expression of immune-related genes in the bursa and spleen of chicks. **(A)** Heatmap of gene expression in the bursa and spleen. In the lower right corner, the purple color indicates low expression change and the red color indicates high expression change. **(B, C)** Immune-related gene expression levels in the bursa and spleen. The different letters (a–c) designated as statistically significant differences (*P<0.05*).

The mRNA expression level of *IFN-γ*, *IL-4*, *IRF7*, *IFIH1*, *TLR3*, *viperin*, and *STAT1* were selected to evaluate the immune response of chicks immunized with CS-gp90@M and CS-gp90@M-M NPs. As shown in **Figure 5**, CS-gp90@M-M significantly upregulated *IFIH1*, *TLR3*, and viperin in the bursa and spleen (*P<* 0.05). The *IFIH1* gene especially was upregulated about 6 times compared to the PBS group (*P*< 0.05). The mRNA expression level of all genes in the CS-gp90@M group except for *IRF7* and *STAT1* group was significantly higher than that in PBS, gp90, and CS-gp90 groups (*P<* 0.05), indicating that the immune response in the CS-gp90@M group was significantly higher than that in the PBS, gp90, and CS-gp90 groups. Taken together, erythrocyte membrane encapsulation and mannose modification promoted immune responses to the nucleic acid vaccine.

### 4.9 Biodistribution of CS-gp90@M-M NPs in chicks

**Figure 6:**
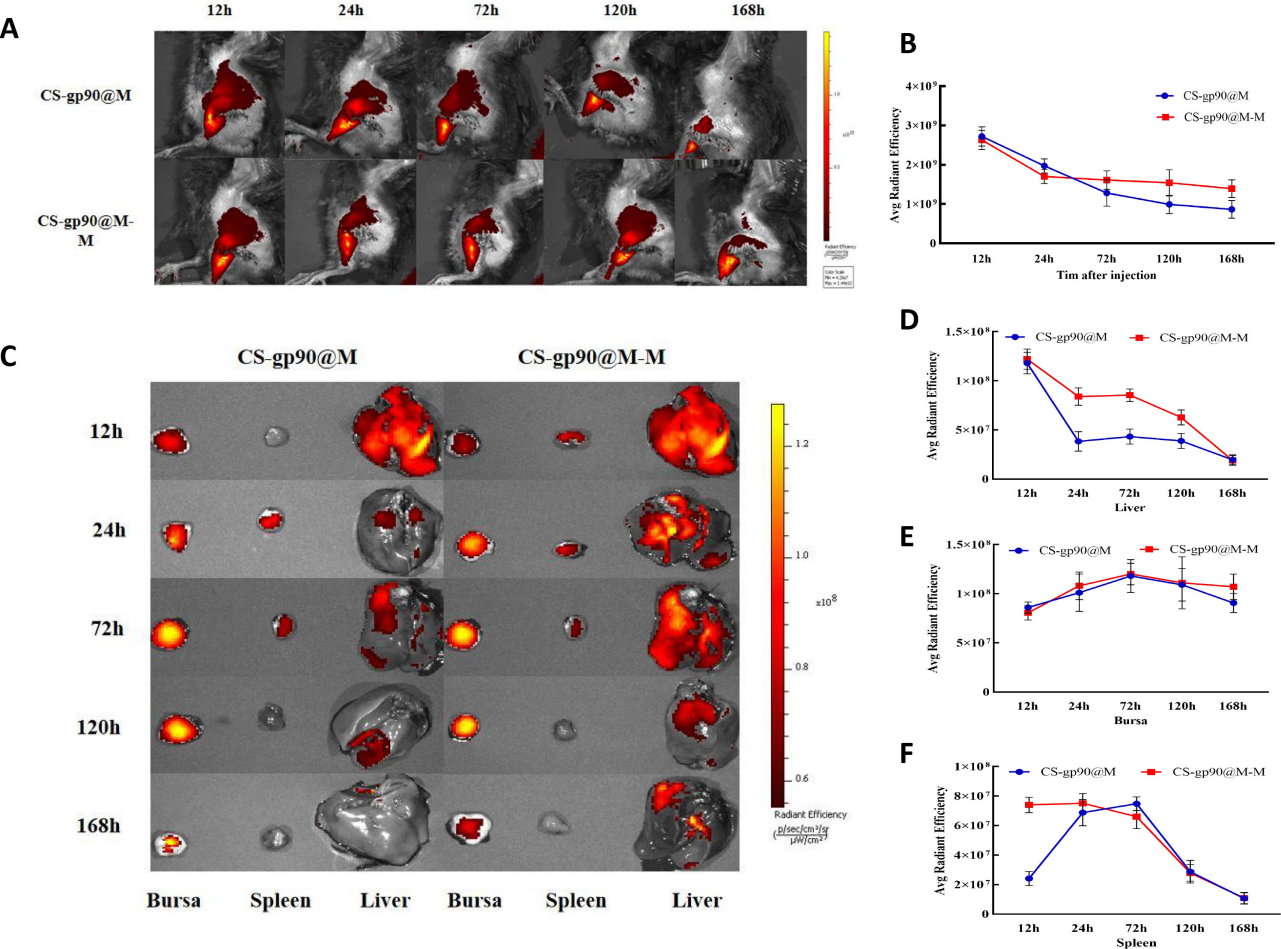
Release and biodistribution of CS-gp90@M-M NPs *in vivo*. **(A, B)** *In vivo* fluorescence images of chicks and the attenuation of the fluorescent dyes over time **(C–F)** Direct imaging of excised organs. Live animal imaging of chicks. The vaccine formulation was stained using a Cy5.5 fluorescent dye and chicks were immunized with CS-gp90@M-M and CS-gp90@M NPs. Live-animal imaging and fluorescence intensity in chicks at 24, 48, 72, and 168 h after injection was determined using an *in vivo* optical imaging system **(A, B)**. Direct imaging and fluorescence intensity of the bursa, spleens, and livers of the injected chicks was determined at 12, 24, 72, 120, and 168 h after injection **(C–F)**.

To determine the release and distribution of the NPs in chicks, CS-gp90@M-M and CS-gp90@M NPs stained with DiD were injected intramuscularly into chicks for immunization. At 24 h after injection, the fluorescence intensity at the injection site was obvious, which then decreased gradually over time. After 24 h, the fluorescence intensity decreased significantly in the chicks in the CS-gp90@M group but decreased slowly in the chicks in the CS-gp90@M-M group (**Figures 6A, B**).

In the case of most vaccines, except for those administered through the gastrointestinal tract, antigens migrate through the APCs to secondary lymphoid organs and are presented to T cells and B cells. After injecting the fluorescence-labeled biomimetic nano-vaccine, fluorescence was first observed in the liver and the intensity decreased gradually as the drug entered the bloodstream (**Figures 6C–F**). After 72 h of inoculation, the fluorescence intensity in the bursa of Fabricius was higher, indicating successful antigen presentation. Antigens that migrated to the lymph nodes were presented to T cells and B cells to further initiate the immune response. Additionally, the fluorescence intensity in the spleens of chicks in the CS-gp90@M-M group was remarkably higher than those in the CS-gp90@M group within 24 h (*P<* 0.05), which may have been related to the targeting effect of mannose. In general, CS-gp90@M-M NPs could induce targeting, prolong the residence time of drugs in the bursa of Fabricius, and help achieve an effective and sustained immune response.

### 4.10 Protective effect of CS-gp90@M-M NPs

**Figure 7:**
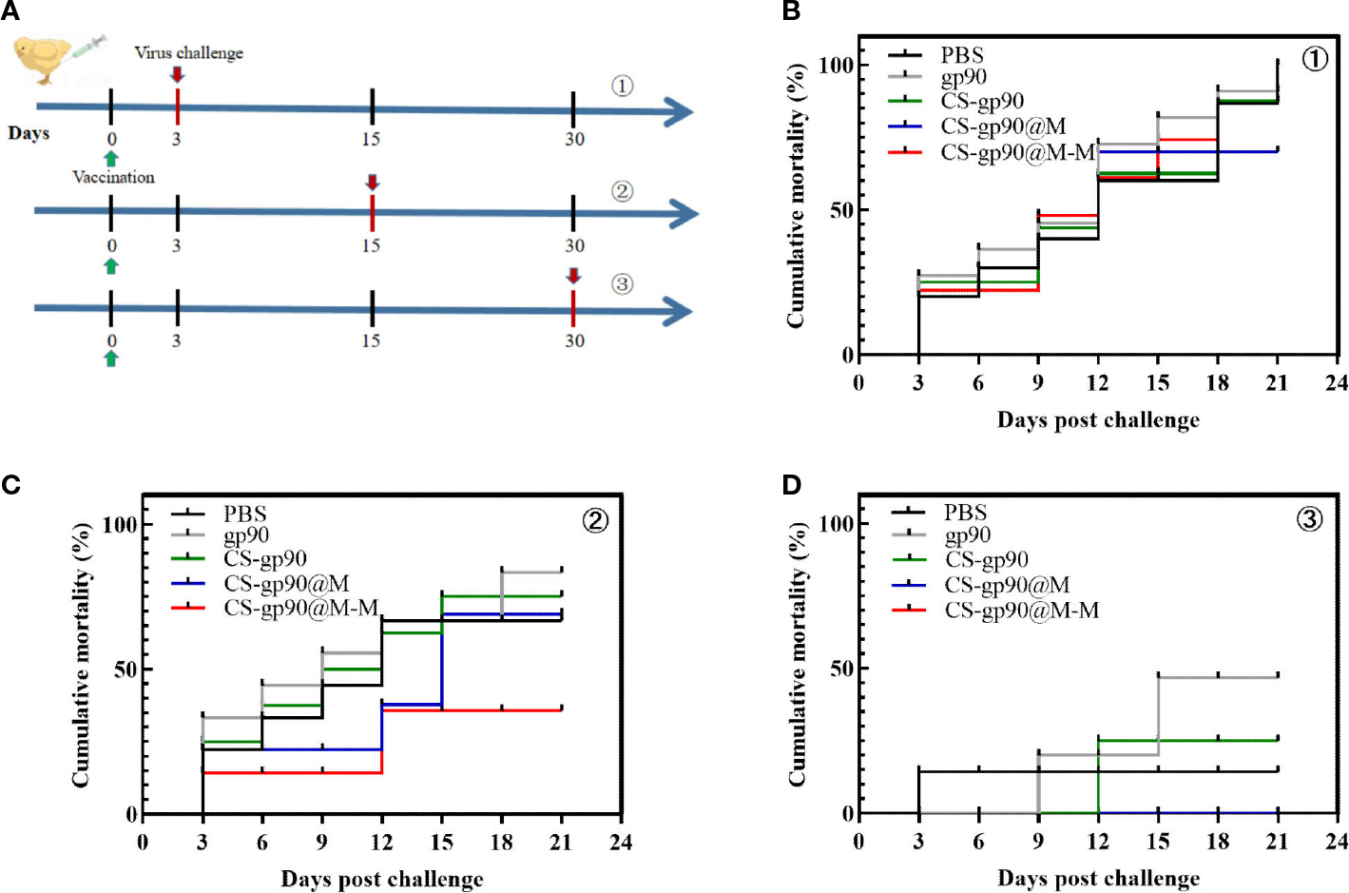
Protective effect of CS-gp90@M-M NPs in chicks infected with REV. **(A)** Schematic diagram demonstrating the REV challenge. Vaccinated chicks were challenged with REV on the 3rd **(B)**, 15th **(C)**, and 30th **(D)** days after vaccination.

As shown in **Figure 7**, the mortality rate was the highest on the 3rd day after vaccination and was related to younger age, undeveloped immune systems, and the weak physique of the chicks. Similarly, on day 30 after vaccination, the mortality in each group was lower, and there were significant differences among the groups only in body weight, although the cumulative mortality of the chicks treated with CS-gp90@M-M NPs was the lowest. The results of the challenge test on day 15 after vaccination were as expected, and the cumulative mortality rate in the CS-G@M-M group was the lowest (20%). In general, the immunoprotective effect of the vaccine was primarily decided by adaptive immunity, which elicited a robust immune response 2 – 3 weeks after vaccination. These findings suggested the CS-gp90@M-M nano-vaccine demonstrated immunoprotective effects against REV in the chicks.

Avian reticuloendotheliosis can induce immunosuppression and lead to growth-inhibition syndrome. The affected chicks usually show growth stagnation and progressive weight loss (31). **Table S2** shows that CS-gp90@M-M NPs greatly improved the body weight of the chicks after the challenge, and CS-gp90@M NPs also improved the body weight of uninfected chicks. Thus, the nano-formulation using erythrocyte membranes and mannose could promote the immunoprotective effect of the nucleic acid vaccine synthesized in this study.

### 4.11 Evaluation of biomimetic nano-vaccine toxicity

**Figure 8:**
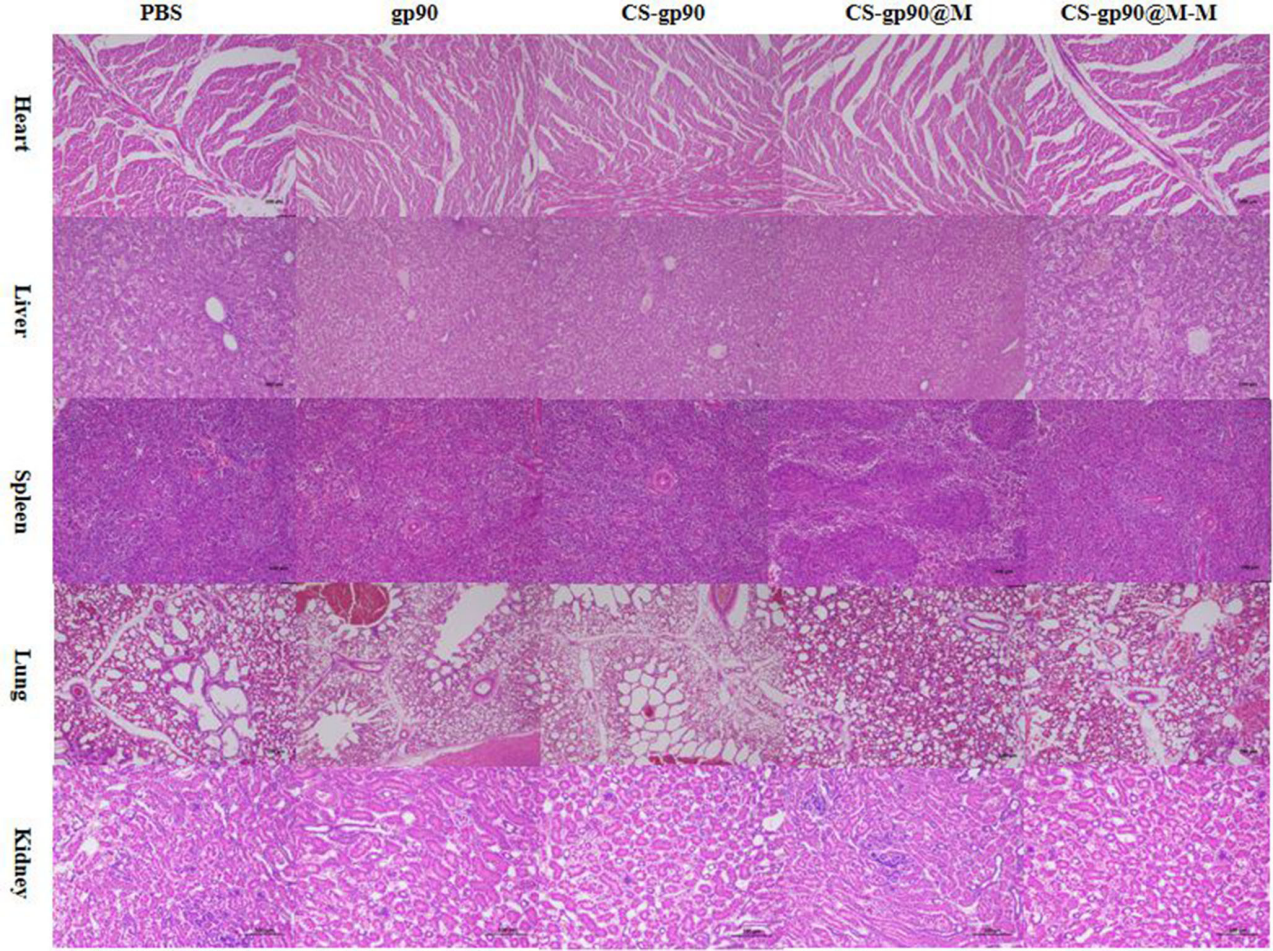
Analysis of potential *in vivo* toxicity. H&E staining of the lungs, heart, spleen, liver, and kidneys of vaccinated chicks on day 28 after immunization. Magnification: 100×, scale bars: 100 μm.

To evaluate the safety of CS-gp90@M-M NPs as vaccine adjuvants, the potential toxicity of CS-gp90@M-M NPs in major organs was evaluated. The heart, lung, liver, spleen, and kidney were sectioned and subjected to H&E staining. Compared to the PBS group, the test group showed normal tissue structures with no significant inflammation or injury (**Figure 8**). The body weight is a crucial biomarker to evaluated the the potential toxicity of CS-gp90@M-M NPs. As illustrated in **Table S3**, Compared to the PBS group, the body weight of chicks in test group showed no significant difference (**Figure 8**). These results indicated that CS-gp90@M-M NPs did not cause distinct toxicity *in vivo*. Therefore, CS-gp90@M-M NPs showed good biocompatibility *in vivo* and demonstrated no toxicity when used as a vaccine-delivery vehicle.

## 5 Discussion

Macrophages are important immune cells in the body and play important anti-infection, anti-tumor, and immune-regulating roles (32). Macrophages uptake pathogens through phagocytosis. The determination of the efficiency of NP phagocytosis by macrophages is a commonly used method to evaluate the immune activity of nano-vaccines. Zhang constructed chitosan-based NPs loaded with spring viremia of carp virus (SVCV) DNA, then encapsulated them with membranes from teleost erythrocytes, and further modified them with a mannose moiety to produce CS-G@M-M NPs. They found that large amount of CS-G@M-M NPs was up took by macrophages (33). In the current study, the intensity of red fluorescence–stained CS-gp90@M-M NPs near the blue nucleus was significantly higher than the intensity of red fluorescence in the CS-gp90@M group. The CLSM image results indicated that more CS-gp90@M-M NPs were internalized by the macrophages. In addition, the quantitative analysis of the uptake of CS-gp90@M and CS-gp90@M-M NPs by macrophages demonstrated that the average fluorescence intensity of HD11 cells incubated with CS-gp90@M-M NPs was higher than that of CS-gp90@M NPs without mannose modification. After modifying CS-gp90@M NPs with mannose, HD11 could phagocytic more antigens. This may have been due to the blinding of mannose residues and mannose receptors on the macrophage surface. The current results demonstrated that grafted mannose significantly improved the macrophage-targeting ability of NPs.

Immunoglobulin (Ig) is a protein produced by B cells and refers to a globulin with antibody (Ab) activity or chemical structure similar to that of antibody molecules (34). Immunoglobulin is a tetrapeptide chain structure composed of 2 identical light chains and 2 identical heavy chains connected by disulfide bonds between the chains. Ig can specifically bind to the corresponding antigen when the antigen stimulates the body’s immune system. Igs are divided into 5 classes, namely, immunoglobulin G (IgG), immunoglobulin A (IgA), immunoglobulin M (IgM), immunoglobulin D (IgD), and immunoglobulin E (IgE). Among these, IgG is the most common, and most antibody reactions involve IgG-mediated effector functions (35). The presence of specific IgG in the serum represents the body’s humoral immunity levels and indicates the body’s immune status. Feng et al. encapsulated ovalbumin (OVA) in poly(lactic-co-glycolic acid) (PLGA) microspheres (MPs) and conjugated them with MAN-modified CS to produce MAN-R-targeting nano-MPs (MAN-CS-OVA-PLGA-MPs). The *in vivo* immune response of the MPs was investigated using a mouse model. The results demonstrated that MAN-CS-OVA-PLGA-MPs significantly increased OVA-specific IgG and IgG isotope antibody levels, suggesting that mannose modification significantly enhanced the humoral immune response of CS-OVA-PLGA-MPs (36). In the present study, the REV-specific IgG antibody titers in the immunized chicks in the CS-gp90@M treated group were significantly increased compared to chicks in the CS-gp90 treatment group, suggesting that erythrocyte membrane coating could promote humoral responses induced by CS-gp90. In addition, compared to the other groups, the serum REV-specific IgG antibody levels in the chicks in the CS-gp90@M-M group were significantly increased after the 2nd week following booster immunization, and higher antibody titers were maintained for a long time, indicating that CS-gp90@M-M NPs could induce higher levels of IgG antibodies than CS-gp90@M NPs. The current results demonstrated that mannose-modified erythrocyte membranes could upregulate the humoral response induced by CS-gp90@M NPs in chicks.

Th1 and Th2-type immune responses can be stimulated by T-cell factors and produce immune responses against pathogens invading host cells. IL-4 is considered a typical cytokine. It is produced by T-helper Th2 cells and is responsible for activating cytotoxic T cells (3, 37). It plays a key role in the development of T and B lymphocytes and in driving humoral immune responses and antibody production (38). IL-4 has an immunomodulatory effect on B cells, T cells, mast cells, macrophages, and hematopoietic cells (4, 39). IFN-γ is a pro-inflammatory cytokine secreted by natural killer cells and activated T cells and is indispensable for adaptive and innate immunity against viral, bacterial, and protozoan infections (12, 40). In the current study, CS-gp90@M-M NPs increased both Th1 and Th2-type cytokine production in immunized chicks, indicating that CS-gp90@M-M NPs significantly induced a balanced Th1/Th2 immune response. The present results also suggested that mannose modification could increase the stimulation of cytokine secretion by CS-gp90@M NPs in chicks and induce both Th1 and Th2-type immune responses.

T lymphocyte subsets play a crucial role in immune modulation and can be divided into Th lymphocytes and suppressor lymphocytes (41). As a marker of Th lymphocytes, CD4^+^ antigens can stimulate B cells to produce antibodies (42, 43). CD8^+^ can reflect the level of cellular immune response (44). In the humoral immune response, there is an increase in CD4+ cell numbers, a decrease in CD8+ cell numbers, and an increase in the CD4+/CD8+ ratio (45). In the present study, CS-gp90@M and CS-gp90@M-M NPs significantly increased the proportion of CD4^+^T cells and the CD4^+^/CD8^+^ ratio, whereas they decreased the proportion of CD8^+^ T lymphocytes. Therefore, both CS-gp90@M-M and CS-gp90@M NP treatments could partially promote the immune response by enhancing T lymphocyte subsets in immunized chicks. The effect of CS-gp90@M-M NPs was more pronounced than that of CS-gp90@M NPs, indicating that mannose-targeted NPs could promote the transmission of bionic NPs in T cell subsets. The present results revealed that mannose-modified erythrocyte membrane coating may be an effective way to improve the efficacy of nano-vaccines in activating T cell subsets in chicks.

During virus infection or vaccine injection in chicks, a series of immune responses occur, and important genes related to immune responses play a key role in the immune response process. IFN-γ and IL-4 are important immunoregulatory cytokines. IFN-γ is a Th1 cytokine, which mainly performs the function of promoting cellular immunity. IL-4 is a Th2 cytokine, which has the primary function of promoting humoral immunity (46). TLR3 is the main receptor in chicks that helps to recognize RNA viruses (47). *IFIH1* are mainly related to the virus response, innate immune response, and the control of apoptosis (17). Viral infections can induce the high expression of *IRF7* (48), and high expression of *STAT1* is observed in response to IFN (49). Viperin is a virus-inhibitory protein (50). In the present study, the mRNA expression level of *IFN-γ*, *IL-4*, *IRF7*, *IFIH1*, *TLR3*, *viperin*, and *STAT1* was determined to evaluate the immune response of chicks immunized with CS-gp90@M and CS-gp90@M-M NPs. The results showed that CS-gp90@M-M NPs significantly increased the expression levels of *IFIH1*, *TLR3*, and viperin in the bursa and spleen. Interestingly, the *IFIH1* gene was upregulated about 6 times compared to the PBS group, indicating that CS-gp90@M-M NPs could induce stronger immune responses in secondary immune organs, which could help to prevent viral infections. The results also demonstrated that the mRNA level of *IFN-γ*, *IL-4*, *IFIH1*, *TLR3*, and *viperin* in the CS-gp90@M group was significantly higher than in the PBS, gp90, and CS-gp90 groups, indicating that erythrocyte membrane encapsulation and mannose modification could promote immune responses to the nucleic acid vaccine. In addition, the upregulated expression of immune-related genes in the spleen was found to be higher than that in the bursa of Fabricius due to the presence of more APCs in the spleen.

The cumulative release properties of an antigen or adjuvant are key features reflecting the function of an antigen delivery system. Live-animal imaging methods are commonly used to analyze the release characteristics of fluorescently labeled antigens delivered from the injection site, and the fluorescence imaging of isolated main organs and tissues can represent the *in vivo* biodistribution of fluorescently labeled NPs at different time points (51). In the current experiment, the fluorescence intensity of DiD-stained CS-gp90@M-M and CS-gp90@M NPs at the injection site was obvious at 12 h and then decreased gradually over time. After 24 h, the fluorescence intensity decreased remarkably at the injection site in the chicks in the CS-gp90@M group but was slowly attenuated in the chicks in the CS-gp90@M-M group.

During the process of vaccination to trigger an immune response, antigens are taken up by APCs, migrate to secondary lymphoid organs and are presented to T cells and B cells. In the present study, the fluorescence intensity of DiD-labeled CS-gp90@M-M and CS-gp90@M NPs was first measured in the liver 12 h after injection and the fluorescence intensity gradually decreased over time. The fluorescence intensity in the bursa of Fabricius was observed from 12 h to 168 h, suggesting that the antigen was successfully presented in secondary immune organs. Antigens that migrated to the lymph nodes were presented to T cells and B cells, to further initiate the immune response. The intensity in the chicks in the CS-gp90@M-M group at 168 h was notably higher than in the CS-gp90@M group, indicating that CS-gp90@M-M NPs had stronger sustained release in immune organs. Furthermore, the fluorescence intensity in the spleens in the CS-gp90@M-M group was remarkably higher than that in the CS-gp90@M group within 24 h, which may have been due to the targeting effect of mannose modification. Taken together, CS-gp90@M-M NPs could achieve targeting, prolong the residence time of drugs in the bursa of Fabricius, and elicit a stronger and sustained immune response compared to the other groups.

An ideal vaccine elicits a robust immune response 2–3 weeks after vaccination, and the determination of the immunoprotective ratio is a method commonly used to assess vaccine immune responses (52). In this study, the mortality rate was the highest on the 3rd day after vaccination. The mortality in each group was lower on day 30 after vaccination, and there were significant differences among the groups only in body weight, although the cumulative mortality of the chicks treated with CS-gp90@M-M NPs was the lowest. The challenge test results on day 15 after vaccination were as expected, and the cumulative mortality rate in the CS-G@M-M group was the lowest (20%). The current data indicated a notable immunoprotective effect in the chicks in the CS-gp90@M-M NP-immunized group given a REV challenge. This may have been due to mannose decorated on CS-gp90@M NPs to increase the immunoprotective effects of this vaccine.

## 6 Conclusions

In summary, for the first time in the field, we investigated the immune response of mannose-modified avian erythrocyte membrane-encapsulated NPs as a novel biomimetic drug delivery system with a characteristic core-shell structure as a biomimetic nano-vaccine. The results showed that bionic encapsulation with erythrocyte membranes and mannose-targeting modification significantly improved the phagocytosis ability of macrophages and induced immune response *in vivo* and *in vitro*. In addition, this chitosan-based novel delivery system could carry plasmid DNA and induce immunoprotective responses after the challenge. Therefore, red blood membranes as a biomimetic targeted modification material enhanced the immune response against REV, and mannose-modified erythrocyte membrane decorating may be an effective strategy for the development of nano-vaccine systems for use against several poultry diseases.

## Data availability statement

The original contributions presented in the study are included in the article/**Supplementary Material**. Further inquiries can be directed to the corresponding author.

## Ethics statement

The animal study was reviewed and approved by Institutional Animal Care and Use Committee of Southwest Minzu University.

## Author contributions

Investigation, HF and YF. Conceptualization, YF and HF. Methodology, YF, ZL and DW. Software, YF. Validation, LZ and QL. Formal analysis, DW. Data curation, QL, SL, DW, FT and HL. Writing—original draft preparation, YF. Writing—review and editing, funding acquisition, project administration, HF. All authors contributed to the article and approved the submitted version.
