# Corrigendum: Mannose-modified erythrocyte membrane-encapsulated chitovanic nanoparticles as a DNA vaccine carrier against reticuloendothelial tissue hyperplasia virus

**DOI:** 10.3389/fimmu.2025.1566389

**Published:** 2025-03-06

**Authors:** Yangyang Feng, Feng Tang, Sheng Li, Daiyan Wu, Qianqian Liu, Hangyu Li, Xinnan Zhang, Ziwei Liu, Linzi Zhang, Haibo Feng

**Affiliations:** ^1^ College of Animal Husbandry and Veterinary Medicine, Southwest Minzu University, Chengdu, China; ^2^ Key Laboratory of Ministry of Education and Sichuan Province for Qinghai-Tibetan Plateau Animal Genetic Resource Reservation and Utilization, Southwest-Minzu University, Chengdu, China

**Keywords:** reticuloendothelial virus bionic, chitosan, erythrocyte membrane, mannose modification, delivery system

In this published article, there was an error in **Figure 3** as published. The incorrect image for group PBS, CS-gp90@M, and CS-gp90@M-M in **Figure 3B** was uploaded by mistake. The corrected **Figure 3** and its caption appears below.

**Figure 3:**
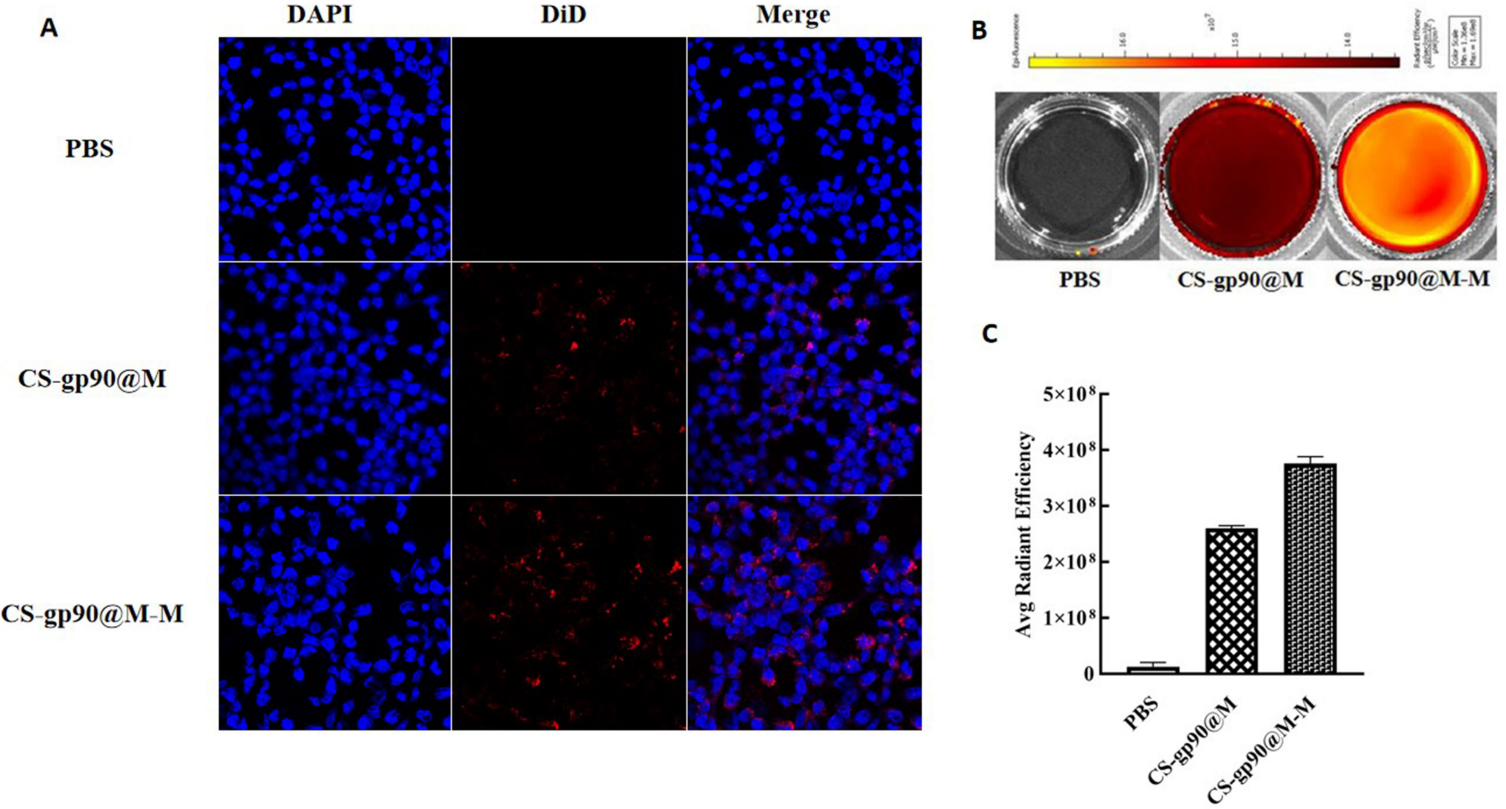
CS-gp90@M-M uptake by macrophages. **(A)** CLSM image of DiD-stained CS-gp90@M and CS-gp90@M-M nanoparticles up take by macrophages *in vitro*. Macrophage solution (8 × 10^4^ cells/mL) was added to a 24-well plate, and 50 μL of DiD-stained CS-gp90@M and CS-gp90@M-M (200 μg/mL) nanoparticles were added and incubated for 8 h. Then, the cells were stained with DAPI dye and washed twice for 20 min. The cells were mounted using glycerol (90%) and observed by CLSM. Blue fluorescence represents DAPI-stained nuclei and red fluorescence represents DID-stained CS-gp90@M and CS-gp90@M-M nanoparticles. **(B)** The uptake of CS-gp90@M-M nanoparticles by macrophages using an IVIS instrument. Macrophages were added to a 6-well plate and cultured for 24 h. DiD-stained CS-gp90@M (50 μL) or CS-gp90@M-M (200 μg/mL) nanoparticles were added to macrophages (5 × 10^5^) in a Petri dish for 8 h. The cells were collected and the intracellular fluorescence intensity of DID was determined using the IVIS instrument. The image shows the fluorescence intensity of the cellular uptake of CS-gp90@M and CS-gp90@M-M nanoparticles by macrophages. **(C)** The average radiant efficiency of the macrophage uptake of nanoparticles.

In this published article, there was an error in the legend for **Figure 6** as published. The incorrect body and organ image for group CS-gp90@M-M at 168h in **Figure 6A** and **Figure 6C** was incorrectly uploaded. The corrected **Figure 6** and its caption appears below.

**Figure 6:**
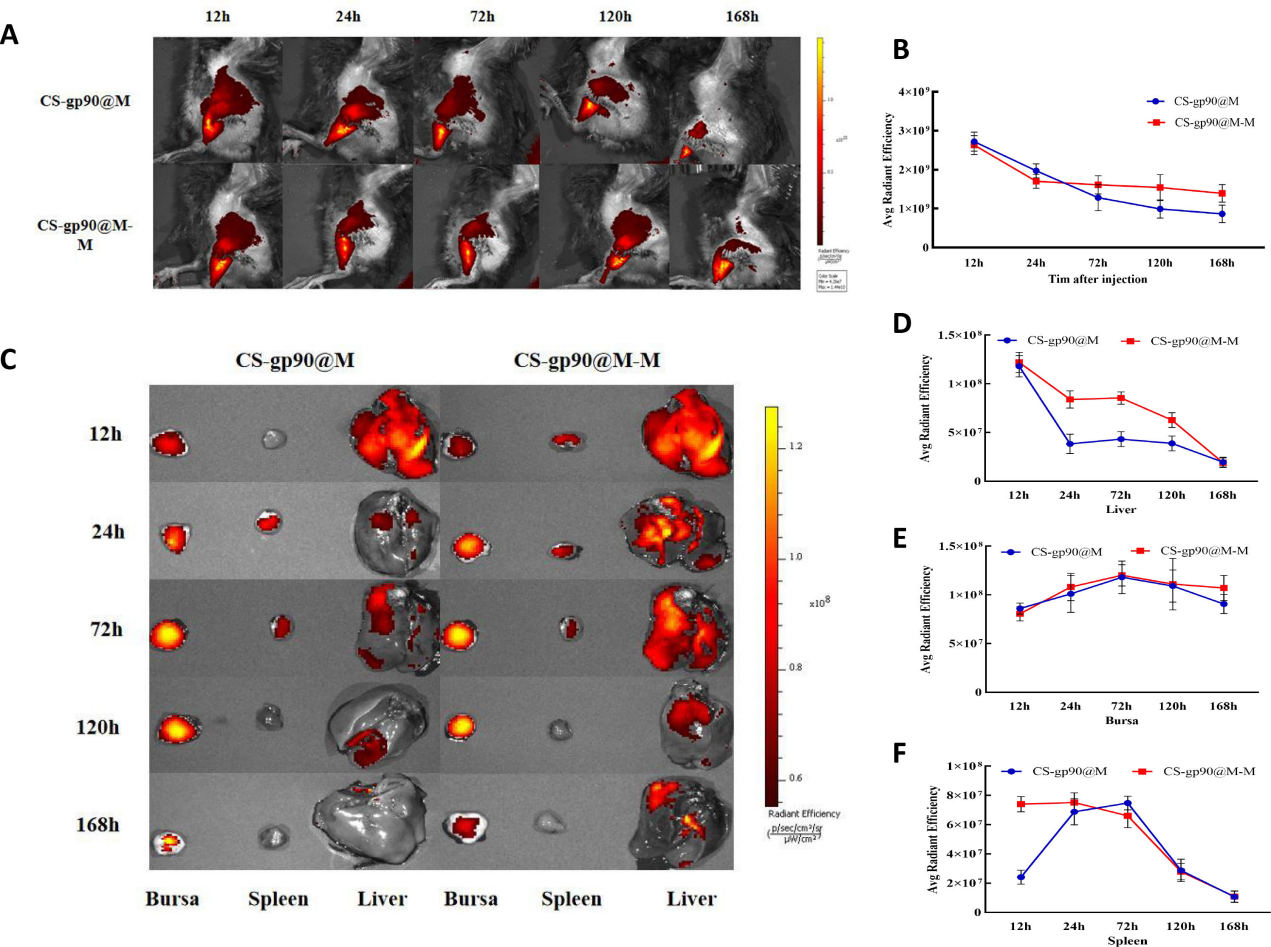
Release and biodistribution of CS-gp90@M-M NPs *in vivo*. **(A, B)** *In vivo* fluorescence images of chicks and the attenuation of the fluorescent dyes over time **(C–F)** Direct imaging of excised organs. Live animal imaging of chicks. The vaccine formulation was stained using a Cy5.5 fluorescent dye and chicks were immunized with CS-gp90@M-M and CS-gp90@M NPs. Live-animal imaging and fluorescence intensity in chicks at 24, 48, 72, and 168 h after injection was determined using an *in vivo* optical imaging system **(A, B)**. Direct imaging and fluorescence intensity of the bursa, spleens, and livers of the injected chicks was determined at 12, 24, 72, 120, and 168 h after injection **(C–F)**.

In the published article, there was an error in **Figure 8** as published. The incorrect use of pathological sections of spleen in the CS-gp90@M group were uploaded in **Figure 8**. The corrected **Figure 8** and its caption appears below.

**Figure 8:**
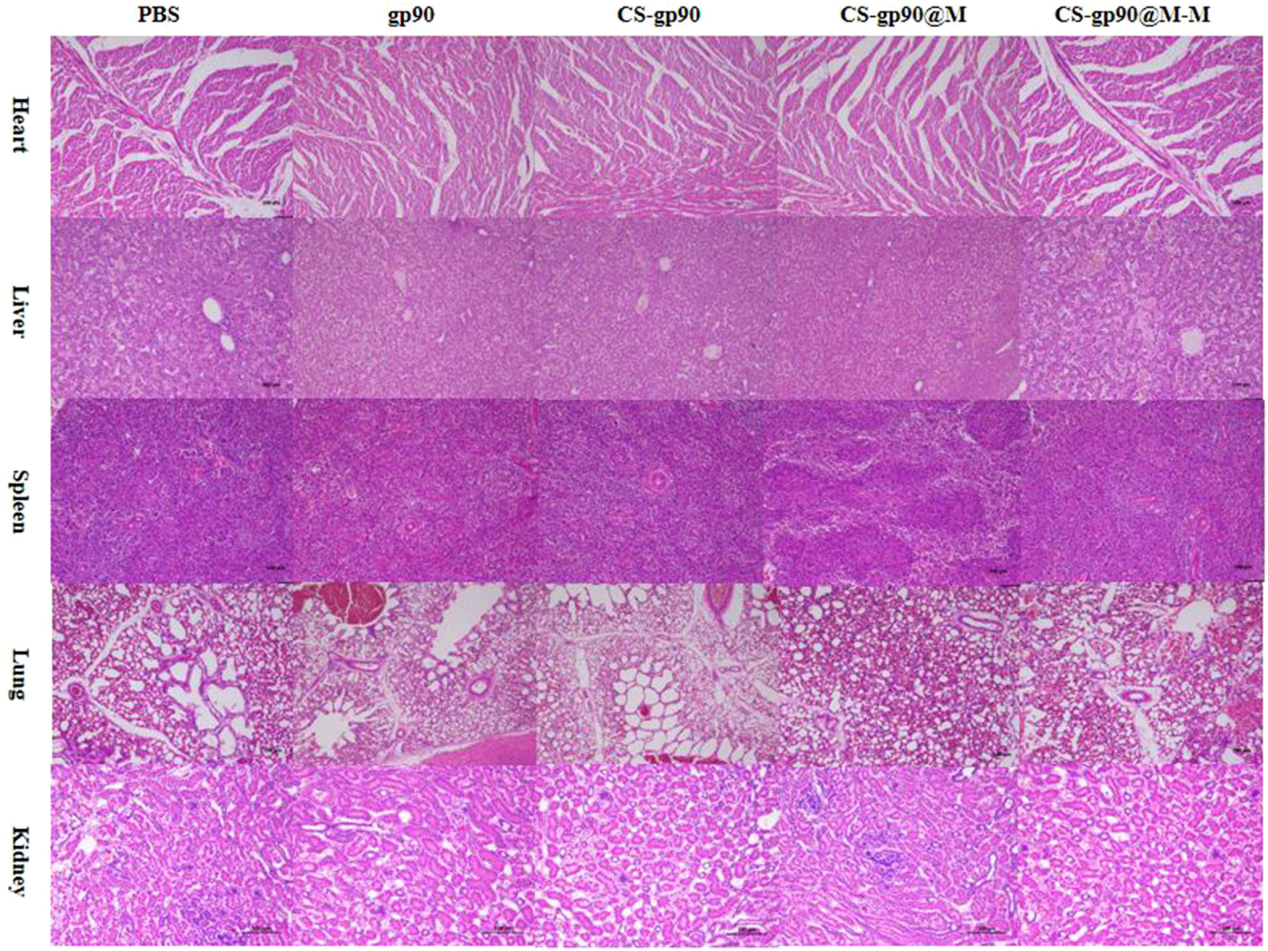
Analysis of potential *in vivo* toxicity. H&E staining of the lungs, heart, spleen, liver, and kidneys of vaccinated chicks on day 28 after immunization. Magnification: 100×, scale bars: 100 μm.

The authors apologize for these errors and state that they do not change the scientific conclusions of the article in any way.

