# Supplementary material for: Mannose-modified erythrocyte membrane-encapsulated chitovanic nanoparticles as a DNA vaccine carrier against reticuloendothelial tissue hyperplasia virus
Source: Front Immunol. 2023 Jan 4;13:1066268. doi: 10.3389/fimmu.2022.1066268 (PMC9910308; doi:10.3389/fimmu.2022.1066268)
Supplement: Supplementary file 1 [file DataSheet_1.doc]

**Table S1 Gene primers**

| Gene name | Primers |
| --- | --- |
| iNOS | F: 5’-GGATTTCCCCAGGCAACCA-3’ |
|  | R: 5’-GCCCAATAGCCACCTTCAGTAC-3’ |
| IL-4 | F: 5’-AGAGGTTTCCTGCGTCAAGATG-3’ |
|  | R: 5’-AGTGCTGGCTCTCCCAAACA-3’ |
| IFN-γ | F: 5’-ATCATACTGAGCCAGATTGTTTCG-3’ |
|  | R: 5’-TCAAGTCGTTCATCGGGAGC-3’ |
| viperin | F: 5’-ACGGTGGTTCAAGAAGTATGGTG-3’ |
|  | R: 5’-CAGCATAATCTCGGCACCACT-3’ |
| TLR3 | F: 5’-GCTTTCAAGAGCCTGAGAACACTA-3’ |
|  | R: 5’-TCCGGGTATATATGCTTGAGTGTC-3’ |
| IFIH1 | F: 5’-AGATTCTGGGACTTACAGCCTCAC-3’ |
|  | R: 5’-AATGGTTCCTTCACCTGATTCTTC-3’ |
| IRF7 | F: 5’-CAAAGCCCAAGGAGTCCAAGC-3’ |
|  | R: 5’-CTGACGTTGCCACTGTTGAGG-3’ |
| STAT1 | F: 5’-GGAAACGGCTACATTAGGACTGA-3’ |
|  | R: 5’-CCATCCGAGATACCTCATCAAACT-3’ |
| β-actin | F: 5’-TGTGCTGTCCCTGTATGCCTCT-3’ |
|  | R: 5’-GGAGGGCGTAGCCTTCATAGA-3’ |

**Table S2 Record of weight changes after challenge**

| Batch | Group | 7 d | 14 d | 21 d |
| --- | --- | --- | --- | --- |
| 3 days after immunization | PBS | 83.45±5.39 | 100.40±9.87 | \ |
|  | gp90 | 82.67±6.62 | \ | \ |
|  | CS-gp90 | 83.86±5.17 | 99.87±10.82 | \ |
|  | CS-gp90@M | 84.29±5.97 | 102.61±12.33 | \ |
|  | CS-gp90@M-M | 85.25±6.22 | 103.89±11.59 | 114.40±15.51 |
| 15 days after immunization | PBS | 243.49±22.57 | 282.96±30.20 | 310.54±35.19 |
|  | gp90 | 236.53±25.39 | 294.17±32.76 | 341.12±33.11 |
|  | CS-gp90 | 245.31±24.41 | 294.49±30.88 | 322.25±34.86 |
|  | CS-gp90@M | 252.65±25.33 | 339.82±33.34 | 398.45±35.34 |
|  | CS-gp90@M-M | 257.45±26.21 | 355.41±32.77 | 423.40±33.39 |
| 30 days after immunization | PBS | 482.43±45.19 | 528.45±43.90 | 566.45±40.03 |
|  | gp90 | 483.29±43.06 | 508.97±44.22 | 552.79±43.92 |
|  | CS-gp90 | 478.01±45.77 | 499.20±43.01 | 543.05±45.76 |
|  | CS-gp90@M | 497.25±45.39 | 527.11±45.98 | 572.21±43.51 |
|  | CS-gp90@M-M | 512.54±42.96 | 583.03±44.04 | 698.09±46.36 |

**Table S3. The body weight of chicks.**

| Group | 0d | 7d | 14d | 21d | 28d |
| --- | --- | --- | --- | --- | --- |
| PBS | 37.36±2.97 | 96.18±8.76 | 198.87±20.57 | 283.74±26.59 | 423.69±39.38 |
| gp90 | 36.58±3.49 | 97.14±10.73 | 196.73±18.04 | 290.40±29.31 | 437.05±40.12 |
| CS-gp90 | 38.15±3.21 | 103.82±11.05 | 202.92±19.59 | 288.73±30.04 | 419.30±41.63 |
| CS-gp90@M | 37.90±3.81 | 101.65±9.90 | 200.39±19.87 | 292.70±31.07 | 442.31±41.05 |
| CS-gp90@M-M | 37.84±4.03 | 98.25±10.73 | 197.84±20.06 | 285.52±29.06 | 434.76±43.44 |

No statistical difference between groups at the same time (p < 0.05).
